# Continuation of the Cases Illustrative of the Practice Pursued at the Royal Westminster Ophthalmic Hospital, and of the New Remedies Latterly Employed in Various States of Disease

**Published:** 1829-01

**Authors:** G. J. Guthrie

**Affiliations:** Professor of Anatomy and Surgery to the Royal College of Surgeons; Surgeon to the Westminster Hospital; and to the Royal Westminster Ophthalmic Hospital.


### THE LONDON
### Medical and Physical Journal.

N° 359, VOL. LXI.] JANUARY, 1829. [N° 31, *New Series.*

For many fortunate discoveries in medicine, and for the detection of numerous errors, the world is indebted to the rapid circulation of Monthly Journals; and there never existed any work, to which the Faculty, in Europe and America, were under deeper obligations than to the *Medical and Physical Journal of London*, now forming a long, but an invaluable series.—RUSH.

### ORIGINAL PAPERS,
AND
### CASES OBTAINED FROM PUBLIC INSTITUTIONS AND OTHER AUTHENTIC SOURCES.

### INFLAMMATION OF THE EYES.

*Continuation of the Cases illustrative of the Practice pursued at the* Royal Westminster Ophthalmic Hospital, *and of the new Remedies latterly employed in various States of Disease.* By G. J. Guthrie, F.R.S. Professor of Anatomy and Surgery to the Royal College of Surgeons; Surgeon to the Westminster Hospital; and to the Royal Westminster Ophthalmic Hospital.

The first three cases show the superiority of the stimulant ointments in disease of long standing, where other remedies appeared to be of little use, or had failed. The eighth, twelfth, and thirteenth show their efficacy in chronic inflammation combined with ulceration. The ninth, tenth, eleventh, and fourteenth prove the utility of the Ung. Argent. Nitrat. in the purulent inflammation of children. The fifth case is a comparative trial of the merits of the two ointments. The fourth and sixth the rapid manner in which a cure is sometimes effected. The fifteenth and seventeenth are comparative trials of different modes of treatment. The twentieth, twenty-first, and twenty-second deserve particular attention, as showing an influence exerted by these remedies they were not in any way suspected to possess. The twenty-third is added as illustrative of the efficacy of an opposite mode of treatment, and to demonstrate the advantage resulting from due discrimination in the treatment of ophthalmic diseases.

Case I.—Margaret Murphy, aged eleven, admitted August 5, 1828. Has had chronic inflammation of both eyes for three years, ending in May last, at which time she was put on the use of the Ung. Argent. Nitrat. At the commencement of the three years, and at intervals afterwards, she attended at the Infirmary, but with little benefit. The eyes were red and painful; she was incapable of bearing the light, and the lids were often almost spasmodically closed. She took medicines of every description, but unavailingly. From the first application of the Argentum Nitratum ointment, she received some relief, which each succeeding one increased, until she was quite well.

The mother is under treatment with the same disease.

Case kept by Mr. GUTHRIE.

CASE II.—Ralph Staton, aged fifty-four, has had bad eyes for three years, proceeding (he thinks) from reading a great deal by candle light; had severe pains in his head at the commencement of the disease, and at different periods of the three years; has suffered frequent violent recurrence of acute inflammation. There is now a lippitudinous affection of the lids; redness of the conjunctivæ, which are villous, and almost granulated; a great discharge of hot water and of a glutinous matter, particularly at night; a nebula on the cornea of the left eye; general health very good. He has had leeches and blisters at different times.

August 9th.—The Ung. Argent. Nitrat. applied to both eyes.

10th.—The smarting from the ointment lasted three-quarters of an hour, after which he could see better than he had done for three months. The discharge greatly diminished.

11th.—Repeat Unguent.

16th.—Absented himself for several days, and the inflammation of the left eye has returned.

C. cum ferro. ad ℥xij. tempori sinistro.—Ung. Arg. Nitr. dextro oculo.

19th.—Better. Repeat ointment to both eyes.

21st.—Rapidly improving. Repeat Unguent.

Discharged cured.—Case kept by Mr. TAYLOR.

CASE III.—June 21st: Francis Westbrooke, aged eighteen, has had sore eyes for seven years, better and worse. Four years ago was under Mr. Guthrie's care, for a year and a half, when he got well, but the disease has gradually returned. At present, can scarcely open either eye, particularly the right, and cannot allow them to be touched; the sensibility is extreme to light, and there is an almost constant spasmodic winking of the lid; the discharge considerable; pain great.

Ung. Argent. Nitrat.

24th.—Repeat. 26th.—Repeat.

July 1st.—Repeat. 3d.—Is much better.

The ointment continued every second or third day, until the 5th of August, when the spasmodic winking of the lid, and the extreme sensibility, were said to be removed.

August 19th.—Nearly well.

Sept. 2d.—Discharged cured.—Case kept by Mr. TAYLOR.

Case IV.—Elizabeth Espenin, aged four; August 23d, 1828. Both eyes have been bad for three months, but they are now worse than ever. Pustules on the cornea of both eyes, with numerous red vessels running from the conjunctivæ to them; great intolerance of light; pain in the head; bowels very irregular; eruptions about the nose and mouth.

℞. Hydr. cum Creta et Rheo gr. x. omni nocte. Infus. Sennæ mane.—Ung. Argent. Nitrat. to both eyes.

24th.—Both eyes nearly well; bowels very much purged.

Omit the Senna. Repeat the powder and the ointment.

25th.—Better. Repeat.

27th.—Could not see on the 22d: is now all but well.

The ointment to be omitted; the purgatives to be continued until the eruption disappears.

Case kept by Mr. Taylor.

Case V.—Edward Riley, nine months old; admitted 17th July with purulent inflammation; was taken ill Saturday, 12th July, the eye appearing weak. On Monday, the 14th, a great watery discharge came on, accompanied by matter; the conjunctivæ very red; the eyelids constantly closed.

17th.—To the left eye, the Ung. Hydr. Oxymur.; to the right, the Ung. Argent. Nitrat.

19th.—Both eyes are better from the application of the ointments, but the right has improved the most.

Repeat the ointments.

21st.—The right eye is nearly well; the left is better; the discharge has nearly ceased; the eyes are both open; the cornea of the left muddy.

The Ung. Argent. Nitr. to the right; nothing to the left.

24th.— Right eye cured.

To the left, the Ung. Hydr. Oxymur.

26th.—Repeat.

29th.—In this case a comparison was instituted between the two ointments, the eyes being nearly similarly affected. The Ung. Hydr. Oxym. did not seem to act as well as the Ung. Argent. Nit. which was therefore directed to be applied to the left.

31st.—The left eye, since the application of the Ung. Argent. Nitr., is considerably better.

Aug. 4, 7, 12, 19.—Repeated the ointment each day, and cured.

Case kept by Dr. Pockrowsky.

Case VI.—Charles Davies, aged sixteen; admitted 16th Aug. Has been ill a month with inflammation and purulent discharge from both eyes; the conjunctivæ of the lids red and swollen, and extending also to that of the globe.

Ung. Argent. Nitrat. to both eyes.

18th.—Declares himself greatly relieved; the pain and uneasiness less, as well as the discharge.—Repeat ointment.

August 19th and 20th.—Did not attend.

21st.—Says he is cured by two applications.

Case kept by Mr. Taylor.

Case VII.—Mary Ann Fensom, aged fifteen; admitted Jan. 16th, 1828. Catarrhal inflammation, which came on Sunday the 14th, with pain in the eye and headache; a running of hot water, and a feeling as if the eyes were full of sand; there is a little muco-purulent discharge, and a general redness of the conjunctivæ.

The Ung. Argent. Nitr. to the left eye; warm water to the right.—Three leeches. Pulv. Jalap. C. ʒ i.

18th.—The left eye, to which the ointment has been applied, is much better; the right eye much the same.

Repeat, and to both eyes.

21st.—Apply the ointment to both eyes.

24th.—Cured; and "deserving of remark." J. Bell.

The progress of the disease was checked after the second application of the ointment. J. B.

Case VIII.—Mary Tuckley, aged four years and a half; admitted 29th July, 1828. Ulcer of the cornea of the left eye, with several large vessels running to it; chronic.

Ung. Argent. Nitrat.

31st.—Much better.

August 1st, 5th, 7th.—Nearly well.

12th.—All trace of inflammation gone: a small white spot marks the place where the ulcer was. J. Bell.

Case IX.—John Clark, five years old; admitted February 28, 1828, with purulent inflammation of both eyes, which is supposed to have been caught from his sister, who is now recovering. Discharge considerable, with great intolerance of light.

Ung. Argent. Nitr. to both eyes.—Hydr. Submur. gr. iv. o. n. Infus. Sennæ mane.

March 2d.—Better. 4th, 7th.—Repeat.

13th.—Discharged cured.

Lives in Manor Gardens, Chelsea.

Case X.—Mary Ann Stockhill, aged seven; admitted March 18th, with purulent inflammation of the lids; the conjunctiva covering the ball not much affected.

Hydrarg. cum Creta gr. vj. o. n.—Lotio Aluminis.

20th.—Is much the same.

Apply the Unguent. Argent. Nitrat. to both eyes.

21st.—Nearly well. 23d.—Cured.

Lives, 8, Brownlow street, Drury lane. Case by Mr. Fernie.

Case XI.—Charlotte English, aged five; admitted April 22d, 1828, with purulent inflammation of the eyelids. Has a sister ill, from whom she is supposed to have caught the complaint. Ill two days. Considerable discharge, and intolerance of light.

Ung. Argent. Nitr. to both eyes.—Hydr. Submur. gr. iij. nocte. Inf. Sennæ mane.

26th.—Repeat.

29th.—Better in every respect, Repeat. May 3d. Well.

Case kept by Mr. Marshall.

Case XII.—Mary Anne Strange, six years old; admitted March 20, 1828. Chronic inflammation of both eyes, with ulceration of the upper part of both corneæ, following small-pox; general muddiness of each.

Ung. Argent. Nitrat.—Inf. Sennæ.

25th.—Very much better. Repeat Inf. Sennæ and ointment.

27th.—Improving fast. Repeat. April 10th.—Ulcers healed.

17th.—Repeat. 29th.—Discharged cured.

Case kept by Mr. Hall.

Case XIII.—Harriet Woodcock; admitted June 5th, 1828, with inflammation of the conjunctivæ and ulceration of the corneæ, after small-pox.

℞. Hydr. Submur. gr. iv. h. somni. Inf. Sennæ mane.—Ung. Argent Nitrat.

June 6th.—Repeat pilula. Magnes. Sulph. ℥ss. mane.

10th.—Improving. Repeat pilula, unguent., et Inf. Sennæ.

12th.—Repeat. 17th.—Repeat. 19th.—Repeat.

24th.—Discharged cured. Case kept by Mr. R. Bell.

Case XIV.— — Falconer, three weeks old; admitted 19th June, 1828, with purulent inflammation, which he has had from his birth. The lids are red and much swollen, with considerable purulent discharge; the child cannot open the eyes.

Ol. Ricini.—Ung. Argent. Nitr. to both.

20th.—The mother says the child is much better.

Repeat Ol. Ricini only.

22d, 24th, 26th, 28th; July 1st, 2d.—Each day the ointment repeated; and, on the 5th, discharged cured.

Case XV.—William Faythorn, aged nine; admitted 16th July, with pustular inflammation of both eyes.

Right eye, apply Ung. Hydr. Oxymur.; left eye, Ung. Argent. Nitr.

12th.—Both ointments have answered exceedingly well, and both have effected a cure by one application.

Case by Dr. Pockrowsky.

Case XVI.—Michael Rooke, aged six; admitted 24th July, 1828, with catarrhal inflammation of the right eye, of four days' standing. Complains of headache and pain in the eye, which is very red, and in a state of considerable chemosis.

Hydr. Subm. gr. iv. Inf. Sennæ.—Ung. Argent. Nitrat.

26th.—The right eye better, but the left was attacked yesterday. The conjunctiva is covered in places by patches of red, like extravasation; the eye is very painful; discharges hot water and matter, and feels, he says, like sand in it.

Apply the Ung. Argent. Nitrat. to both.

29th.—Discharged cured. Case by Mr. R. D. Mitchell.

Case XVII.—Thomas Elwick, aged eight; admitted January 1, 1828, with inflammation and ulcers of the cornea.

Three leeches. Calomel gr. iij. o. n. Inf. Sennæ. Empl. Lyttæ.

3d.—Repeat the leeches, pill, and senna.

8th.—Better, and to continue the remedies.

15th, 22d.—The inflammation having subsided, and becoming apparently chronic, the ulceration remaining stationary, the Solut. Argent. Nitrat., four grains to the ounce, to be applied. The pills and senna to be repeated.

February 1st.—Much the same.

9th.—Stationary: repeat pill and senna.

19th.—If any thing, rather worse.

21st.—To be cupped behind each ear to two ounces.

Hydr. cum Creta et Rheo gr. viij. bis die.

28th.—Has gradually become worse: cannot open the eyes, which are very irritable, and the intolerance of light is great; discharge cold.

The Ung. Argent. Nitrat. to be well applied to both eyes, and no other remedy.

March 2d.—Is very much better; has opened both eyes, and can look about him, bearing the light tolerably well.

Repeat the ointment.

6th.—Much better. March 12th.—Discharged cured.

Lives, 28, Gue lane, Kensington.

Case XVIII.—Joseph Catlin, aged ten; admitted May 13th, 1828. A pustule in the centre of the cornea, with great inflammation and discharge of hot tears. Has had a sore eye for three years, but not so bad as it has been for the last week.

℞. Hydr. Submur. gr. iv. statim. Inf. Sennæ vespere. Empl. Lyttæ pone aurem. Cucurb. cum ferro ad ℥v.

14th.—Inflammation less; the cupping gave great relief; he can open his eye better. Repet^r medicamenta.

15th.—Improving.

Hydr. Sub. nocte. Magnes. Sulph. ℥iv. mane. Aqua tepida.

20th.—Cured.

On the 27th, returned, having, as his mother said, caught cold, and relapsed; the inflammation being as bad as at first, the child looking pale and ill.

Apply the Ung. Argent. Nitrat.

29th.—Apply the ointment. Much better.

Returned the 17th June, saying he got well after the last application of the ointment, and has now been bad four days; the inflammation considerable, and the cornea more opaque in the centre than formerly. Apply the ointment.

19th,—Better. 21st.—Repeat. 24th.—Repeat.

July 8th.—Repeat. Pil. Hydr. Subm. gr. iv. Inf. Sennæ.—Unguent. Argent. Nitrat.

10th.—Repeat. 16th.—Discharged cured.

Case by Dr. POCKROWSKY.

CASE XIX.—Mary Anne Flood, three years old; admitted 28th June, 1818. Disease of a week standing, but with little or no pain. The iris discoloured and inflamed, but no lymph effused on the edge of the pupil, which is regular, although it seems furred on the surface around it; considerable sclerotic inflammation, with ulcer of the cornea; lymph deposited at the under part of the cornea, forming an onyx.

To be cupped to three ounces from the temple. Hydr. Subm. gr. ij. every two hours. The Ung. Hydr. fort. ʒss. to the forehead daily.—Inf. Sennæ.

29th.—Much relieved by the cupping.

30th.—Better: repeat medicines.

July 1st.—Much better. To continue the pills and the ointment in smaller quantities, although the pills have not been regularly given.

5th.—Omit the medicines, the internal inflammation having subsided. The mouth has not been sore, but the child is weakened, and looks pale.

17th.—Quite well, with the exception of a very slight speck.

Case by Mr. CAVIE.

CASE XX.—Charles Campion, twenty-one years old; admitted February 7th, 1828. Sclerotic inflammation.

To be cupped on the temple to twenty ounces. Hydr. Subm. gr. vj. Magnes. Sulph. ℥j. each night.

10th.—A great deal better. Repeat the pills and salts.

12th.—The inflammation is now more confined to the tunica conjunctiva; the tears run hot, and there is intolerance of light.

The Unguent. Argent. Nitr. to be applied.—Pulv. Jalap. C. ʒiss.

14th.—Much better. The ointment and powder continued to the 14th March, when he was discharged cured.

Case by Mr. BELL.

CASE XXI.—William Malhuish, aged twenty-two; admitted September 16th, 1828. Had a chancre five months ago, which was cured under a course of mercury. The eye has been bad a month: has had lotions applied, and mercury given, which he has only left off taking ten days; the mouth is yet sore. Has had eruptions all over his body. No pain in the eye, but some in the forehead; dull red sclerotic inflammation; pupil irregular, and larger than natural. Mr. Guthrie said this was a return of inflammation in the vessels of the sclerotica and iris, from weakness, on the application of cold.

The Unguent. Argent. Nitrat.

18th.—Better: repeat. 20th.—Repeat.

23d.—Very much better; the pupil more regular, and diminished in size; lymph going.

25th.—Nearly well. 27th.—Cured. Case by Mr. Taylor.

Case XXII.—William M'Owen, aged twenty-four; admitted May 15th, 1828, with inflammation of the iris of the left eye, of a low kind; little or no pain; pupil irregular; no lymph thrown out; a dark coloured zone of vessels around the cornea.

The Ung. Argent. Nitrat. applied. Six grains of the Submuriate of Mercury at night, and one ounce of Sulphate of Magnesia in the morning.

17th and 20th.—Repeated.

22d.—Pulv. Jalapæ C. ʒi. Solutio Belladonnæ, the inflammation having subsided.

25th, 28th.—Repeat.

June 3d.—The right is affected in a similar manner: sclerotic inflammation, loss of transparency of the cornea, the pupil irregular, vision diminished, the iris discoloured.

Ungent. Argent. Nitrat. alone, to ascertain its effect.

5th.—The whole complaint is better, the inflammation being very much diminished, the cornea clearer; the pupil still irregular; vision the same.

Repeat the ointment.

7th.—Says he was better yesterday than this day, but is still better than on the 5th.

Repeat the ointment.

10th.—A slight irregularity of the pupil is the only remaining appearance of disease.

Apply the Solutio Belladonnæ.

On the 19th July, one month from his discharge, he returned with the following symptoms: There is a zone of red vessels around the cornea, which is muddy, or has lost its transparency; the iris is discoloured, and red vessels may be easily seen running upon it; the pupil is contracted, irregular, and a network of lymph may be seen behind it. The spots of lymph, of a reddish colour, and of the size of pins' heads, have been effused at the under and outer margin of the iris. Little intolerance of light; pain in the forehead and side of the head, increased at night; is covered with lichen syphilitica. Says he had never had sores on the penis, but had a discharge for a few days six months ago.

℞. Hydrarg. Submur. gr. iij.; Ext. Opii gr. ¼, fiat pilula, tertia quaque horâ sumenda.

21st.—Repeat. 23d.—Inflammation subsiding.

24th.—Mouth sore: repeat Hydr. Subm. and apply Belladonna.

26th.—Mouth very sore: omit medicine.

27th.—The Belladonna to be applied every day. The disease nearly gone, the irregularity of pupil only remaining in a trifling degree; the lichen syphilitica disappearing also.

Case XXIII.—James Heavy, aged twenty-six; admitted May 6th, 1828; says he cannot see out of his right eye; thinks he lost the sight of it from sleeping in a house which was then being painted, in November last; no pain, no inflammation. Attended an hospital two months without benefit; he then applied to a gentleman conversant in the ophthalmogistic art, who told him nothing could be done for him; he then came here. Is a stout healthy man, of a sanguineous temperament.

To be bled from the jugular vein to sixteen ounces.—Pil. Calomel gr. vj. nocte. Magnes. Sulph. ℥i. mane.

10th.—To be bled from the arm to twelve ounces.

Repeat pill and salts.

12th.—Repeat pill and salts. 15th.—Repeat.

17th, 20th.—Can now see at intervals.

To be cupped to twelve ounces; to take two grains of Calomel every night, and half an ounce of Sulphate of Magnesia in the morning.

24th.—To be cupped again to sixteen ounces, the last cupping having done good.

27th.—Empl. Lyttæ tempori. Repetantur Pil. et Magn. Sulph.

29th.—Rep$^r$ Pil. et Magnes. Sulph. 31st.—Repeat.

June 5th.—Has nearly recovered his sight.

12th.—Does not attend regularly, but, when he does come, gets his pill and salts.

July 8th.—Cupped to sixteen ounces.

11th.—Repeat the cupping. 16th.—As well as usual.

August 14th.—Has absented himself for some time, and has returned not so well.

To be cupped to sixteen ounces on the temple.

19th.—Empl. Lyttæ to the neck. Pulv. Jalap. C. ʒiss. statim.

20th.—The powder to be repeated every second day.

28th.—To be cupped to twelve ounces.

September 2d.—Can now see very well, although not quite as well as with the other eye.

Sp. Rosmar occasionally, and Pulv. Jalapæ C.

---

